# The complete mitochondrial genome and phylogenetic position of the Endangered red-spotted grouper *Epinephelus akaara* (Perciformes, Serranidae) collected in South Korea

**DOI:** 10.1080/23802359.2016.1247666

**Published:** 2017-01-05

**Authors:** Yoo-Kyung Kim, Young-Don Lee, Hong-Shik Oh, Sang-Hyun Han

**Affiliations:** a Marine and Environmental Research Institute, Jeju National University, Jeju, South Korea;; b Faculty of Science Education, Jeju National University, Jeju, South Korea;; c Educational Science Research Institute, Jeju National University, Jeju, South Korea

**Keywords:** Endangered species, *Epinephelus akaara*, phylogenetic position, red-spotted grouper

## Abstract

We determined complete nucleotide sequences of the mitochondrial genome of two individuals of the Red-spotted grouper, *Epinephelus akaara* (Perciformes, Serranidae), caught in South Korea. The mitochondrial genome had 16,795 base pairs (bp) and 13 protein-coding genes, 2 ribosomal RNAs, 22 transfer RNAs, and a noncoding control region. The two mt genomes were highly homologous (99.71% similarity). The two mt genomes of *E. akaara* analyzed in this study were found in Clade I with those of *E. awoara*, *E. fasciatomaculosus*, *E. sexfasciatus*, *E. diacanthus*, *E. sticus*, and *E. morio*. Here, we reported the complete mt genome sequence of *E. akaara,* suggesting that this may use in phylogenetic studies of *Epinephelus*.

The Red-spotted grouper, *Epinephelus akaara*, is a western Pacific fish species and designated as Endangered species. This species inhabits in Korea, southern Japan, Taiwan, and southern China, and it generally inhabits shallow coral and rocky reefs.

Genomic DNAs were extracted from two individuals (specimen IDs. BukbariG01and BukbariG05) of *E. akaara*, one collected from the South Sea and the other, from a shallow coral area of Jeju Island, South Korea. The complete mitochondrial (mt) genome was sequenced (accession nos. KJ700439 and KJ700440). Both mt genome sequences of *E. akaara* had 16,795 base pairs and consisted of 37 genes. The two mt genomes were highly homologous (99.71% similarity).

The phylogenetic relationships of *Epinephelus* spp. were examined using mt 13 protein-coding genes of 21 species previously reported in *Epinephelus*. The phylogenetic tree showed three distinct clades (Clade I–III, Figure 1). The present mt genomes of *E. akaara* were found in Clade I with those of *E. awoara*, *E. fasciatomaculosus*, *E. sexfasciatus*, *E. diacanthus*, *E. sticus*, and *E. morio*. Clade II and III contained all other species used in the phylogenetic tree. Molecular studies including *E. akkara* was also described by some researchers (Ding et al. 2006; Han et al. 2014; Zhuang et al. 2013). Here, we reported the complete mt genome sequence of *E. akaara*. This phylogenetic sequence may explain its specific position in the family Epinephelidae. 

**Figure 1.**
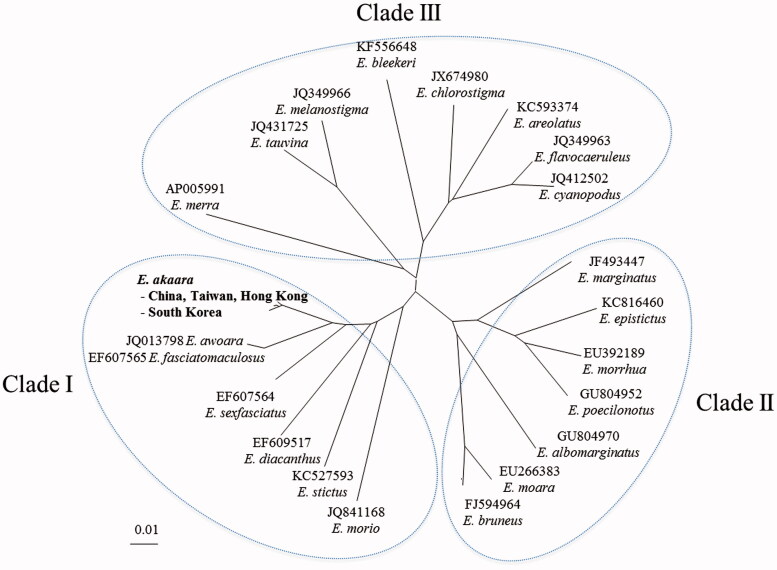
Molecular phylogenetic analysis of mt 13 protein-coding genes in 22 species of *Epinephelus*. The phylogenetic tree was constructed using neighbour-joining method based on the genetic distances general time reversible model as the best nucleotide substitution model based on AICc criterion. The rate variation model allowed for some sites to be evolutionarily invariable. The number above bar indicates genetic distance.
